# CT-assessed sarcopenia is a predictive factor for both long-term and short-term outcomes in gastrointestinal oncology patients: a systematic review and meta-analysis

**DOI:** 10.1186/s40644-019-0270-0

**Published:** 2019-12-03

**Authors:** Huaiying Su, Junxian Ruan, Tianfeng Chen, Enyi Lin, Lijing Shi

**Affiliations:** 1 Department of Radiology, Quanzhou First Hospital Fujian, Quanzhou, Fujian Province People’s Republic of China; 2 Department of Ultrasonic, Quanzhou Women’s and Children’s Hospital, 700 Fengze Road, Quanzhou, 362000 Fujian Province People’s Republic of China; 3 Department of Radiology, Quanzhou Women’s and Children’s Hospital, Quanzhou, Fujian Province People’s Republic of China; 4 Department of Radiology, The First Affiliated Hospital of Fujian Medical University, Fuzhou, Fujian Province People’s Republic of China

**Keywords:** Sarcopenia, Gastrointestinal oncology, Nutrient, Operation

## Abstract

**Background:**

The impact of sarcopenia on the outcome of gastrointestinal (GI) oncological patients is still controversial. We aim to discuss the prevalence of sarcopenia and its relation to the oncological outcome.

**Methods:**

Embase, Medline, PubMed, and the Cochrane library were systematically searched for related keywords. Studies using CT to assess sarcopenia and evaluate its relationship with the outcome of GI oncological patients were included. Long-term outcomes, including overall survival and disease-free survival, were compared by hazard ratios (HRs) with 95% confidence intervals (CIs). Short-term outcomes, including total complications and major complications (Clavien-Dindo ≥IIIa) after curable surgery, were compared by the risk ratio (RR) and 95% CI.

**Results:**

A total of 70 studies including 21,875 patients were included in our study. The median incidence of sarcopenia was 34.7% (range from 2.1 to 83.3%). A total of 88.4% of studies used skeletal muscle index (SMI) in the third lumbar level on CT to define sarcopenia, and a total of 19 cut-offs were used to define sarcopenia. An increasing trend was found in the prevalence of sarcopenia when the cut-off of SMI increased (β = 0.22, 95% CI = 0.12–0.33, *p* < 0.001). The preoperative incidence of sarcopenia was associated both with an increased risk of overall mortality (HR = 1.602, 95% CI = 1.369–1.873, *P* < 0.001) and with disease-free mortality (HR = 1.461, 95% CI = 1.297–1.646, *P* < 0.001). Moreover, preoperative sarcopenia was a risk factor for both total complications (RR = 1.188, 95% CI = 1.083–1.303, P < 0.001) and major complications (RR = 1.228, 95% CI = 1.042–1.448, *P* = 0.014).

**Conclusion:**

The prevalence of sarcopenia depends mostly on the diagnostic cut-off points of different criteria. Preoperative sarcopenia is a risk factor for both long-term and short-term outcomes.

## Introduction

The incidence of gastrointestinal (GI) malignancy is almost 30% worldwide, with high cancer-related mortality [1, 2]. Aging is one of the most significant risk factors for the incidence and mortality in malignancy, usually with an exponential increase [2, 3]. Although there is great development in oncological treatment, surgical resection is still the main curable method [4]. However, for elderly oncologic patients, the incidence of postoperative complications still needs attention due to the nutrition status and potential comorbidities [5].

Sarcopenia was first proposed by Rosenberg in 1989 and was defined as a disease of skeletal muscle mass decline with age and was previously referred to as age-related sarcopenia [6, 7]. The incidence of sarcopenia is 20% in healthy people under 70 years of age, and its incidence is more than 50% after age 80 [8]. An epidemiological survey found that the incidence of muscle reduction in healthy elderly Chinese was 4.1–11.5%. A Japanese epidemiological study found that 14.2% of men and 22.1% of women in the elderly age range had muscle reduction [9]. There are many causes of sarcopenia, such as skeletal muscle disuse, endocrine changes, chronic consumptive diseases, systemic inflammatory response, insulin resistance, and malnutrition [10, 11]. GI cancer is often accompanied by an eating disorder and vomiting, coupled with increased metabolic consumption in the oncological condition, and the probability of malnutrition is higher. Therefore, the incidence of muscle reduction in patients with CRC is significantly higher than that in healthy people, reflecting that the tumor is one of the causes of sarcopenia [12]. Additionally, sarcopenia is a predictor of adverse outcomes in malignant tumors. Several studies have shown that muscle reduction is closely related to the incidence of postoperative complications and the overall survival of esophagus, gastrointestinal tract, hepatobiliary and pancreatic malignancies [13–16]. However, the impact of sarcopenia on the outcome of GI cancer patients remains controversial due to the heterogeneity of different studies, and negative results have been found in different populations [17, 18]. Thus, we designed this systematic review and meta-analysis to examine the prevalence of computed tomography (CT)-assessed sarcopenia in GI oncological patients and therefore discuss the relationship between sarcopenia and long-term and short-term outcomes in GI oncological patients.

## Methods

This study was designed based on the preferred reporting items for systematic review and meta-analysis (PRISMA) guidelines [19].

### Search strategy

A systematic review and meta-analysis were designed to evaluate CT-assessed sarcopenia in predicting the outcomes of gastrointestinal oncology patients. The Embase, Ovid Medline, Cochrane Database of Systematic Reviews and Cochrane Central Register of Controlled Trials and PubMed were systematically searched up to March 25, 2019. In addition, the gray literature was searched using the related websites and Google Scholar. The keywords were designed by experienced librarians. Briefly, the key words included “sarcopenia”, “muscle mass”, “body composition” and “gastrointestinal”, “gastric”, “colorectal”, and “neoplasm”, “lesion”, “tumor”, “cancer” in Mesh and keywords. The search strategy is attached in appendix 1. All the studies containing abstracts and titles were imported into Endnote X6 to find duplicate studies and then for literature screening.

### Inclusion and exclusion criteria

All the studies using CT-assessed sarcopenia or body composition in predicting long-term or short-term outcomes in GI oncology treatment patients were included in our study. The inclusion criteria were as follows: 1) the body composition was assessed by CT; 2) the study had a clear definition for sarcopenia or body composition, with a specific cut-off; 3) the outcome data and clinical data of GI oncological patients could be extracted; 4) GI oncology included esophageal, gastric, intestinal, and colorectal tumors; 5) the study mentioned one or more oncological treatments, such as surgery, chemotherapy, and radiation; 6) the study design was limited to randomized control trials, prospective or retrospective cohort studies, and case-control studies. Meta-analyses, reviews, conference abstracts and comments were read to find more papers. Only the studies written in English were included in the systematic review.

The exclusion criteria were as follows: 1) animal experiments; 2) body composition or sarcopenia assessed by other methods rather than CT; 3) no specific definition or cut-off of sarcopenia or body composition; 4) no available data of outcomes or the prevalence of sarcopenia in GI oncology patients; 5) cancer located in other organs rather than GI systems, such as liver, pancreas, and bladder; and 6) case reports or non-English publications. Data from the same center were treated as one dataset for further meta-analysis.

### Literature screening and data extraction

Two investigators (H.Y.S. and T.F.C.) independently screened the abstracts and titles according to the inclusion and exclusion criteria. The full text was further evaluated if the abstract was not definitive. The third investigator (J.X.R.) was consulted for discussion if any disagreement existed.

A standard Excel spreadsheet was designed for data extraction, and the following information was collected from the original studies: the study characteristics (author, publish year, country, institution, recruitment period, study design, etc.), patient characteristics (location of cancer, treatment, total sample, median age, sex distribution, tumor stage, etc.), assessment approach of sarcopenia or body composition (modality, CT-specific index, definition and cut-off of index), sarcopenia prevalence and outcome assessment (complication rate after surgery, toxicity and progression rate after adjuvant therapy, overall survival and disease-free survival after treatment). The complication after surgery was evaluated based on the Clavien-Dindo criteria, and a major complication was defined as stage IIIa or higher [20].

### Quality assessment

Two reviewers (E.Y.L. and L.J.S.) independently assessed the quality of the included papers. For case-control and cohort studies, the Newcastle-Ottawa Scale (NOS) was used to evaluate the quality. High quality was defined as a score greater than 7, and moderate quality was defined as a score between 5 and 7 [21]. Moreover, the Grading of Recommendations, Assessment, Development, and Evaluation (GRADE) system was used to evaluate the overall quality of the evidence [22].

### Assessment approach of body composition

The CT-quantified muscle mass area was used to assess the sarcopenia. Different criteria, including the skeletal muscle index (SMI), which calculated the area of total skeletal muscle (cm^2^) in the third lumbar (L3) level divided by the height squared (m^2^), total psoas area (TPA), and visceral fat volume and area (VFV, VFA), were commonly used to describe the nutritional status of patients.

### Statistical analysis

The statistical analysis was performed by Stata 15.0 software (Stata Corporation, College station, TX, USA). The prevalence of sarcopenia in different studies was drawn in bubble plots, with the relative sample as the bubble size. Linear trends were analyzed using weighted least squares regression using prevalence as the dependent variable and cut-off of SMI in females as the independent variable with sample size as the weight. The complications were compared and combined using relative risk (RR), while the survival analysis was combined using hazard ratio (HR). Both were reported with a 95% confidence interval (CI), and a *P* value less than 0.05 was set as significant. The I^2^ statistic and χ^2^ test were used for heterogeneity assessment (I^2^ ≥ 50% indicating the presence of heterogeneity). When heterogeneity existed, the random-effect model was used, while the fixed-effect model was used otherwise. Finally, forest plots were drawn, and funnel plots were used to evaluate the publication bias.

## Results

### Literature selection

**Fig. 1:**
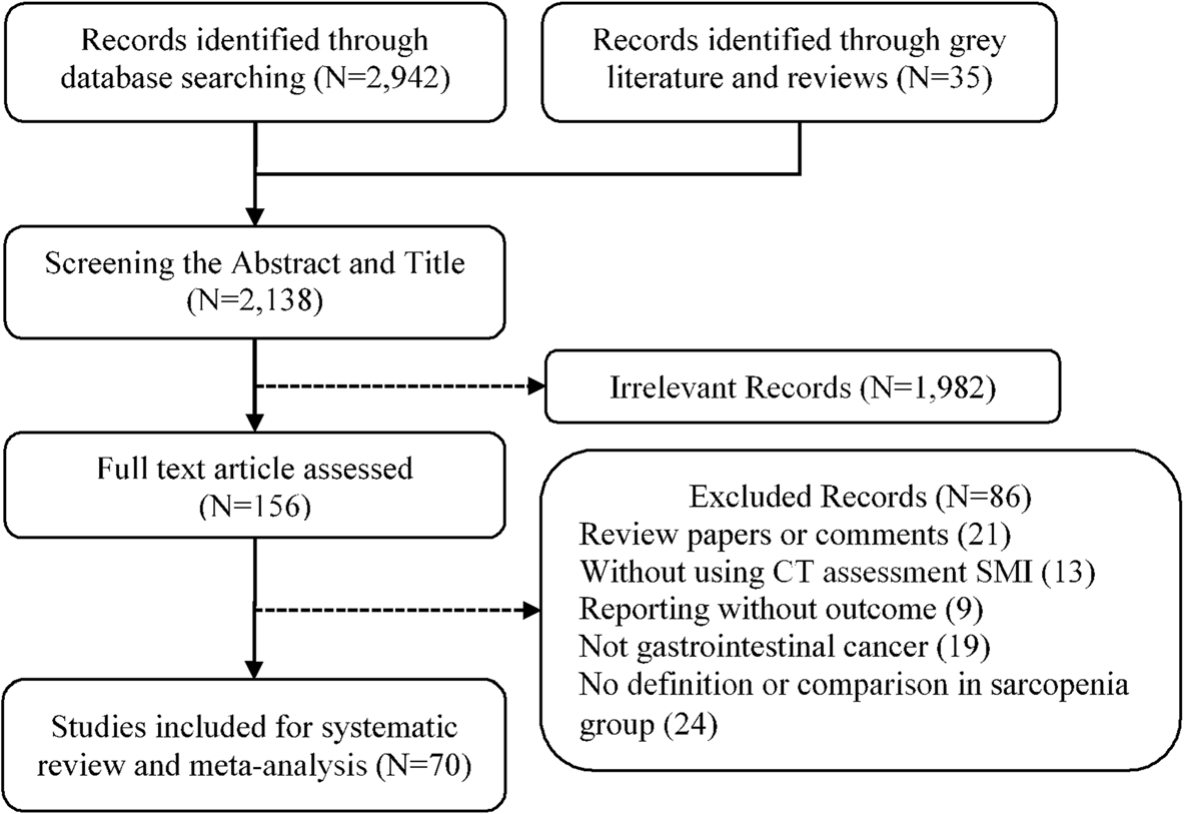
Flowchart of included studies

A total of 2942 studies were found according to the search strategy. The flowchart is shown in Fig. 1. After screening the abstracts and titles, 156 studies were scanned in full. After excluding the incompatible studies, a total of 70 studies were included in the systematic review [6, 13–15, 17, 18, 23–86].

### Characteristics of the included studies

The characteristics of the included studies are shown in Table 1. The first study using body composition to predict the outcomes after treatment in GI oncological patients was published in 2010 [77], while the first study using SMI to define sarcopenia was published in 2012 [75]. Sixty-two studies were retrospective, and eight were prospective, with a recruitment period between 2001 and 2017. A total of 21,875 patients were involved in the systematic review: 1996 esophageal cancer (EC) patients (14 studies), 7913 gastric cancer (GC) patients (27 studies) and 11,875 CRC patients (29 studies). Twelve studies enrolled advanced oncological patients who only received adjuvant treatment, while fifty-seven studies involved patients who underwent surgery combined with adjuvant treatment or not, and the percentage of adjuvant treatment prior or after surgery ranged from 4 to 100%. The median age was 64.6 years (range from 53 to 76 years), and the percentage of male patients ranged from 38 to 92%. The prevalence ranges of tumor stages I, II, and III were 2.78–46.39%, 10.63–56.49%, and 16.12–89.36%.

**Table 1:** Characteristics of included studies

Author	Year	Recruitment period	Design	Disease	Treatment	Total sample	Median age, year	Tumor stage (AJCC, I/II/III/IV)	Male, n (%)	Adjuvant therapy, n (%)
Yang, J.	2019	2011–2017	Retrospective	CRC	Surgery	417	57.9	80/190/149/0	251 (60)	–
Hopkins, J. J.	2019	2007–2009	Retrospective	CRC	Surgery	968	65.8	100/374/494/0	589 (61)	503 (52)
van Vugt, J. L. A.	2018	2007–2013	Retrospective	CRC	Surgery	816	–	255/293/269	440 (54)	158 (19)
van der Kroft, G.	2018	2012–2013	Retrospective	CRC	Surgery	63	–	18/13/20/12	39 (62)	–
Mosk, C. A.	2018	2013–2015	Retrospective	CRC	Surgery	251	76	–	141 (56)	26 (10)
Mauricio, S. F.	2018	2013–2016	Retrospective	CRC	Surgery	84	61.6	36/48 ^a^	39 (46)	51 (61)
Martin, L.	2018	2013–2015	Retrospective	CRC	Surgery	210	66.6	385/713/887/109	1270 (60)	–
Chen, W. Z.	2018	2014–2017	Retrospective	CRC	Surgery	376	64.3	65/155/145/11	228 (61)	–
Feliciano, E. M. C.	2017	2006–2011	Prospective	CRC	Surgery	247	63	690/806/956/0	1251 (51)	–
Black, D.	2017	2006–2014	Retrospective	CRC	Surgery	339		58/153/128/0	181 (53)	66 (19)
Ouchi, A.	2016	2012–2015	Retrospective	CRC	Surgery	60	69	42/18 ^a^	35 (58)	–
Malietzis, G.	2016	2006–2013	Retrospective	CRC	Surgery	805	69	189/265/267/84	472 (59)	182 (23)
Reisinger, K. W.	2015	2010–2012	Retrospective	CRC	Surgery	310	–	–	155 (50)	–
Park, B. K.	2015	2005–2012	Retrospective	CRC	Surgery	543	–	185/314 ^a^	311 (57)	51 (9)
Miyamoto, Y.	2015	2005–2010	Retrospective	CRC	Surgery	220	–	77/84/59/0		54 (25)
Huang, D. D.	2015	2014–2015	Retrospective	CRC	Surgery	142	62	–	88 (62)	5 (4)
Lieffers, J. R.	2012	2002–2006	Retrospective	CRC	Surgery	234	63	0/74/83/77	135 (58)	–
Pedziwiatr, M.	2016	2014–2015	Retrospective	CRC	Surgery	124	65.9	32/32/39/21	73 (59)	–
Jones, K. I.	2015	2011–2012	Retrospective	CRC	Surgery	100	68.6	–	60 (60)	–
Guinan, E. M.	2018	2014–2016	Retrospective	EC	Surgery	27	–	–	–	27 (100)
Mayanagi, S.	2017	2004–2013	Prospective	EC	Surgery	66	63.3	0/27/39/0	57 (86)	66 (100)
Elliott, J. A.	2017	2010–2015	Retrospective	EC	Surgery	207	61.6	–	165 (80)	207 (100)
Black, D.	2017	2006–2014	Retrospective	EC	Surgery	108	–	30/43/35/0	74 (69)	65 (60)
Nishigori, T.	2016	2005–2014	Retrospective	EC	Surgery	199	–	33/99/63/6	164 (82)	–
Grotenhuis, B. A.	2016	2001–2012	Retrospective	EC	Surgery	120	62	–	88 (73)	120 (100)
Yip, C.	2014	NG	Retrospective	EC	Surgery	35	63	0/10/23/2	30 (86)	35 (100)
Nakashima, Y.	2018	2004–2014	Retrospective	EC	Surgery	341	–	38/46/55/33	289 (85)	–
Paireder, M.	2017	2006–2013	Retrospective	EC	Surgery	130	61.4	15/22/76/3	106 (82)	130 (100)
Tamandl, D.	2016	2006–2013	Retrospective	EC	Surgery	200	63.9	45/33/95/4	151 (76)	–
Harada, K.	2016	2005–2011	Retrospective	EC	Surgery	325	–	129/45/128/23	298 (92)	–
Tan, B. H.	2015	2010–2012	Retrospective	EC	Surgery	89	65.8	21/27/41/0	67 (75)	89 (100)
Zhang, Y.	2019	2015–2017	Retrospective	GC	Surgery	156	59.1	48/27/81/0	115 (74)	35 (22)
Zhang, W. T.	2018	2014–2016	Prospective	GC	Surgery	636	–	203/140/293/0	478 (75)	–
Wang, S. L.	2018	2009–2013	Retrospective	GC	Surgery	859	64	239/193/427/0	672 (78)	–
Park, H. S.	2018	2006–2009	Retrospective	GC	Surgery	136	55	0/57/79/0	96 (71)	63 (46)
O’Brien, S.	2018	2008–2014	Retrospective	GC	Surgery	56	68.4	18/13/18/0	41 (73)	28 (50)
Nishigori, T.	2018	2005–2013	Retrospective	GC	Surgery	177	–	0/100/77/0	127 (72)	127 (72)
Mao, C. C.	2018	2014–2016	Prospective	GC	Surgery	682	64.6	–	513 (75)	–
Lin, J.	2018	2015–2016	Prospective	GC	Surgery	670	65	–	–	–
Choi, M. H.	2018	2007–2009	Retrospective	GC	Surgery	98	–	–	–	–
Beuran, M.	2018	2014–2016	Retrospective	GC	Surgery	78	–	6/28/41/13	–	–
Zhou, C. J.	2017	2014–2015	Retrospective	GC	Surgery	240	73	74/55/111/0	190 (79)	–
Zheng, Z. F.	2017	2009–2013	Retrospective	GC	Surgery	639		–	525 (82)	408 (64)
Lou, N.	2017	2014–2015	Retrospective	GC	Surgery	206	64.1	80/45/81/0	161 (78)	–
Kudou, K.	2017	2005–2016	Retrospective	GC	Surgery	148	–	–	–	–
Huang, D. D.	2017	2014–2015	Retrospective	GC	Surgery	470	65	163/103/204/0	364 (77)	–
Zhuang, C. L.	2016	2008–2013	Retrospective	GC	Surgery	937	64	271/219/447	730 (78)	–
Wang, S. L.	2016	2014–2015	Prospective	GC	Surgery	255	65.1	81/48/126/0	190 (75)	–
Takeuchi, M.	2016	2009–2015	Retrospective	GC	Surgery	75	–	25/16/28/6	57 (76)	3 (4)
Huang, D. D.	2016	2014–2015	Prospective	GC	Surgery	173	72	53/40/80/0	135 (78)	–
Tegels, J. J.	2015	2005–2012	Retrospective	GC	Surgery	152	69.6	42/27/47/57	87 (57)	71 (47)
Li, X. T.	2015	2005–2008	Retrospective	GC	Surgery	84	57	0/31/53/0	60 (71)	–
Sakurai, K.	2017	2007–2013	Retrospective	GC	Surgery	569	66.7	264/121/126/58	396 (70)	91 (16)
Chen, F. F.	2016	2014–2016	Prospective	GC	Surgery	158	66.9	33/37/88/0	126 (80)	–
Takeda, Y.	2018	2004–2011	Retrospective	RC	Surgery	144	–	0/45/99/0	102 (71)	63 (44)
Park, S. E.	2018	2005–2015	Retrospective	RC	Surgery	65	71	8/24/27/0	46 (71)	65 (100)
Choi, M. H.	2018	2009–2013	Retrospective	RC	Surgery	188	61.3	0/34/154/0	117 (62)	188 (100)
Heus, C.	2016	2006–2013	Retrospective	RC	Surgery	74	64	–	39 (53)	–
Souza, B. U.	2018	2015–2016	Retrospective	CRC	All	197	60.5	54/138 ^a^	112 (57)	–
Kurk, S. A.	2018	NG	Prospective	CRC	AT	450	–	–	285 (63)	n/a
Chemama, S.	2016	2008–2010	Retrospective	CRC	AT	97	53	–	37 (38)	n/a
Blauwhoff-Buskermolen, S.	2016	2011–2014	Retrospective	CRC	AT	67	66.4	–	42 (63)	n/a
Barret, M.	2014	NG	Retrospective	CRC	AT	51	65	–	38 (75)	n/a
Guiu, B.	2010	2002–2008	Retrospective	CRC	AT	120	–	–	55 (46)	n/a
Anandavadivelan, P.	2016	2006–2012	Retrospective	EC	AT	72	–	2/20/50/0	–	n/a
Awad, S.	2012	NG	Retrospective	EC	AT	47	–	–	34 (72)	n/a
Sugiyama, K.	2018	2013–2015	Retrospective	GC	AT	118	64	–	59 (50)	n/a
Palmela, C.	2017	2012–2014	Retrospective	GC	AT	47	68	0/5/42/0	32 (68)	n/a
Mirkin, K. A.	2017	2000–2015	Retrospective	GC	AT	41	–	–	–	n/a
Hayashi, N.	2016	2009–2014	Retrospective	GC	AT	53	–	–	–	n/a
Nipp, R. D.	2018	2011–2015	Retrospective	GIC	AT	103	–	–	–	n/a

*Abbreviation*: *EC* esophageal cancer, *GC* gastric cancer, *GIC* gastrointestinal cancer, *CRC* colorectal cancer, *RC* rectal cancer, *AT* adjuvant or neo-adjuvant therapy, *NG* not given, *n/a* not available

^a^Tumor stage I and II versus III and IV

### Sarcopenia definition, assessment of prevalence

**Fig. 2:**
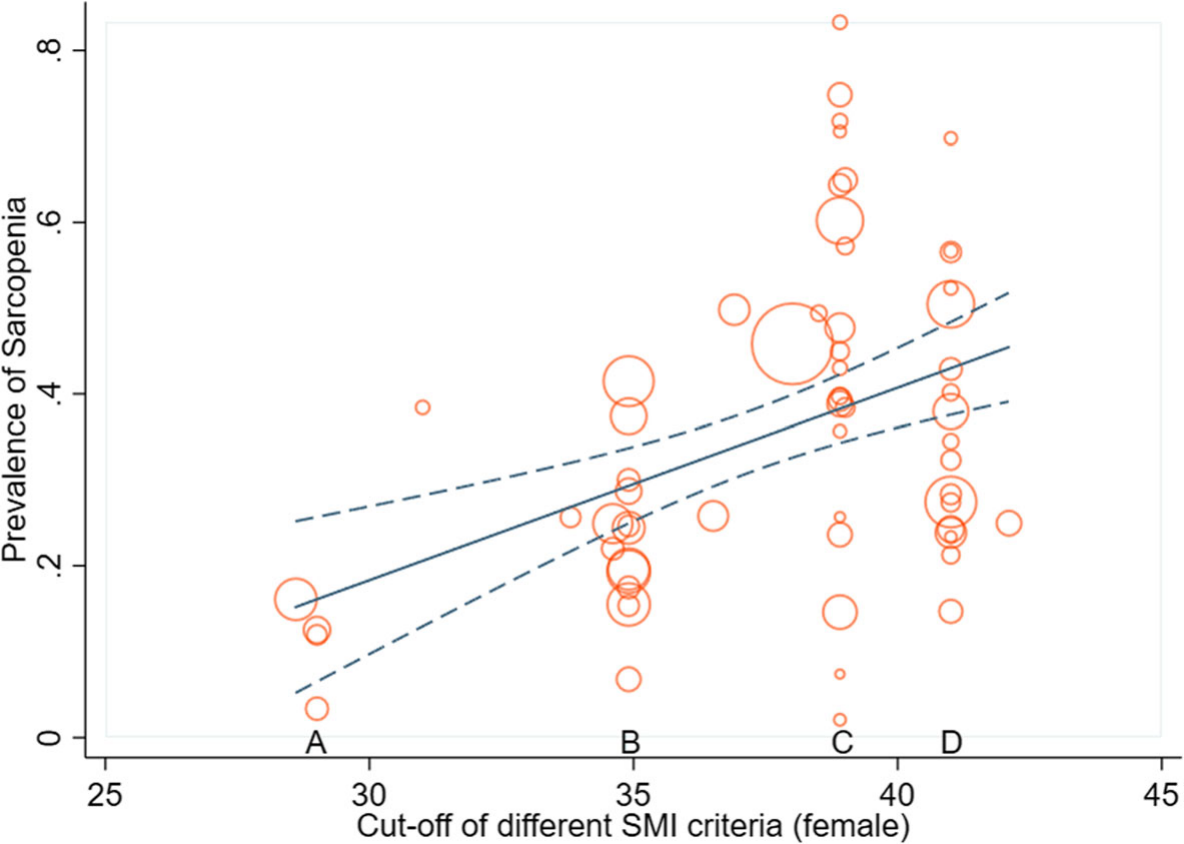
The bubble plots and linear relationship between the prevalence of sarcopenia and the cut-offs for females under different criteria (A. cut-off from Iritani et al.; B. cut-off from Zhuang et al.; C. cut-off from Prado et al.; D. cut-off from Martin et al.)

The studies were mainly from Asia, Europe, North America, and South America, including 15 counties (Austria, Brazil, Canada, China, France, Ireland, Japan, Korea, Netherland, Poland, Portugal, Romania, Sweden, UK and USA). The common cut-offs for evaluating the sarcopenia are listed in Table 2. The median incidence of sarcopenia was 34.7% (range from 2.1 to 83.3%). The majority of studies (88.4%) used SMI in L3 to assess sarcopenia, five studies used visceral fat criteria, and three studies used TPA criteria. Among the studies using SMI, three main criteria were the most commonly adopted criteria, including 47 studies. The cut-off of SMI introduced by Prado et al. in 2008 (sarcopenia was defined as SMI < 52.4 cm^2^/m^2^ for males and SMI < 38.5 cm^2^/m^2^ for females) was used in 20 studies covering 10 countries [23, 30, 34, 35, 43–45, 47, 52, 57, 61, 62, 64, 67, 69, 73–76, 85]. The prevalence of sarcopenia ranged from 7.4 to 83.3% (7.4–71.8% in non-Asian countries, with a median prevalence of 40.1%; 14.6–83.3% in Asian countries, with a median prevalence of 52.7%). The cut-off provided by Martin et al. in 2013 (sarcopenia was defined as SMI < 41 cm^2^/m^2^ in females; SMI < 53 cm^2^/m^2^ if BMI ≥ 25 kg/m^2^ and SMI < 43 cm^2^/m^2^ if BMI < 25 kg/m^2^ in males) was used in 17 studies covering 9 Asian and non-Asian countries [6, 24, 28, 31, 33, 35, 37, 38, 42, 50, 54, 58, 65, 66, 68, 81, 82]. The prevalence of sarcopenia ranged from 14.7 to 69.8% (14.7–56.7% in non-Asian countries, with a median prevalence of 35.1%; 28.4–69.8% in Asian countries, with a median prevalence of 43.4%). The cut-off introduced by Zhuang et al. was generally used in Asian countries (12 studies: [13, 25, 26, 35, 40, 41, 46, 48, 53, 55, 63, 84]), which defined sarcopenia as SMI < 40.8 cm^2^/m^2^ in males and SMI < 34.9 cm^2^/m^2^ in females, but the majority of the studies were from the same center. The prevalence ranged from 6.8–41.5%, with a median of 23.1%. The cut-off provided by Iritani et al. was used in three studies (SMI < 36 cm^2^/m^2^ in male; SMI < 29 cm^2^/m^2^ in female) with a median prevalence of 9.3% [35, 59, 72], and the cut-off provided by Voron et al. was also used in three studies (SMI < 55 cm^2^/m^2^ in male; SMI < 39 cm^2^/m^2^ in female) with a median prevalence of 53.6% [36, 79, 80]. Two other Japanese studies adopted the cut-off from Sakurai et al. (SMI < 43.2 cm^2^/m^2^ in males; SMI < 34.6 cm^2^/m^2^ in females) and had a median prevalence of 23.5% [18, 35]. The prevalence of sarcopenia is plotted in Fig. 2, and an increasing trend was found in the prevalence of sarcopenia as the cut-off of SMI increased (β = 0.22, 95% CI = 0.12–0.33, *p* < 0.001, r^2^ = 0.2170).

**Table 2:** Sarcopenia definition, assessment and prevalence

No	Modality	Index	Cut-off, Male	Cut-off, Female	Method	Prevalence	Reference	Country	NOS
1	CT/L3	SMI	32.5	28.6	Cut-off from 3-year overall survival	16.1%	Zheng, Z. 2017	China	7
2	CT/L3	SMI	36	29	Cut-off from Iritani et al.	3.4%	Nishigori, T. 2018	Japan	7
						12.5%	Wang, S. 2016	China	7
						12.0%	Huang, D. 2015	China	8
3	CT/L3	SMI	40.8	34.9	Cut-off from Zhuang et al.	15.4%	Zhang, Y. 2019	China	7
						19.7%	Zhang, W. 2018	China	6
						17.5%	Nishigori, T. 2018	Japan	7
						19.4%	Mao, C. 2018	China	6
						15.5%	Lin, J. 2018	China	5
						24.5%	Chen, W. 2018	China	7
						28.8%	Zhou, C. 2017	China	7
						6.8%	Lou, N. 2017	China	7
						37.4%	Huang, D. 2017	China	7
						41.5%	Zhuang, C. 2016	China	8
						30.1%	Huang, D. 2016	China	7
						24.7%	Chen, F. 2016	China	6
4	CT/L3	SMI	43.2	34.6	Cut-off from Sakurai et al.	22.0%	Nishigori, T. 2018	Japan	7
						25.0%	Sakurai, K. 2017	Japan	8
5	CT/L3	SMI	44.5	36.5	Cut-off from the third quartile cases	25.8%	Harada, K. 2016	Japan	6
6	CT/L3	SMI	45	33.8	Cut-off from the third quartile cases	25.7%	Takeda, Y. 2018	Japan	7
7	CT/L3	SMI	47.2	36.9	Cut-off from the median of SMI	49.9%	Nakashima, Y. 2018	Japan	8
8	CT/L3	SMI	49	31	Cut-off from Kim et al.	38.5%	Park, S. 2018	Korea	6
9	CT/L3	SMI	49.5	42.1	Cut-off from the third quartile cases	25.0%	Miyamoto, Y. 2015	Japan	7
10	CT/L3	SMI	52.4	38.9	Cut-off from Prado et al.	14.6%	Yang, J. 2019	China	6
						35.7%	O’Brien, S. 2018	Ireland	7
						64.4%	Nishigori, T. 2018	Japan	7
						7.4%	Guinan, E. 2018	Ireland	4
						39.4%	Choi, M. 2018	Korea	7
						39.8%	Choi, M. 2018	Korea	6
						71.8%	Beuran, M. 2018	Romania	5
						83.3%	Mayanagi, S. 2017	Japan	7
						23.7%	Elliott, J. 2017	Ireland	6
						74.9%	Nishigori, T. 2016	Japan	7
						60.2%	Malietzis, G. 2016	UK	7
						45.0%	Grotenhuis, B. 2016	Netherlands	8
						43.1%	Anandavadivelan, P. 2016	Swede	5
						47.7%	Reisinger, K. 2015	Netherlands	6
						25.7%	Yip, C. 2014	UK	5
						38.9%	Lieffers, J. 2012	Canada	7
						2.1%	Awad, S. 2012	UK	4
						49.4%	Tan, B. 2015	UK	5
							Sugiyama, K. 2018	Japan	5
11	CT/L3	SMI	55.4	38.9	Cut-off from Prado et al.	70.6%	Barret, M. 2014	France	5
12	CT/L3	SMI	55	39	Cut-off from Voron et al.	57.3%	Nipp, R. 2018	USA	4
						38.5%	Paireder, M. 2017	Austria	8
						65.0%	Tamandl, D. 2016	Austria	7
13	CT/L3	SMI	43/53 (BMI lower or higher than 25)	41	Cut-off from Martin et al.	27.5%	Hopkins, J. 2019	Canada	7
						50.5%	van Vugt, J. 2018	Netherlands	8
						52.4%	van der Kroft, G. 2018	Netherlands	6
						14.7%	Souza, B. 2018	Brazil	4
						32.4%	Park, H. 2018	Korea	6
						42.9%	Nishigori, T. 2018	Japan	7
						24.3%	Mosk, C. 2018	Netherlands	6
						34.5%	Mauricio, S. 2018	Brazil	7
						38.0%	Kurk, S. 2018	Netherlands	4
						23.4%	Palmela, C. 2017	Portugal	4
						28.4%	Kudou, K. 2017	Japan	8
						21.3%	Black, D. 2017	UK	7
						23.9%	Black, D. 2017	UK	7
						40.2%	Chemama, S. 2016	France	6
						56.7%	Blauwhoff-Buskermolen, S. 2016	Netherlands	6
						56.6%	Tegels, J. 2015	Netherlands	6
						27.4%	Pedziwiatr, M. 2016	Poland	7
						69.8%	Hayashi, N. 2016	Japan	6
14	CT/L3	SMI	52/54 (BMI lower or higher than 30)	38/47 (BMI lower or higher than 30)	Cut-off from Caan et al.	45.9%	Feliciano, E. 2017	USA	6
15	CT/L3	SMI	–	–	z-score below - 0.5 for SMI in different ages	6.7%	Martin, L. 2018	Canada	6
16	CT/L3	TPA	538	346	normal TPA in the lowest sex-specific quartile	33.3%	Ouchi, A. 2016	Japan	6
17	CT/L3	TPA	545	385	Cut-off from Fearon et al.	29.3%	Mirkin, K. 2017	USA	5
						15.0%	Jones, K. 2015	UK	4
18	CT	VEV	1.92	1.92	Cut-off from the quartiles	25.0%	Park, B. 2015	Korea	5
19	CT	VFA	100	100	Cut-off from the Japanese Society for study of Obesity	35.9%	Wang, S. 2018	China	7
						65.3%	Takeuchi, M. 2016	Japan	6
						40.5%	Heus, C. 2016	Netherlands	4

*Abbreviation*: *CT/L3* the third lumbar vertebra level in CT scan, *BMI* body mass index, *SMI* skeletal muscle index, *TPA* total psoas muscle area, *VEV* visceral fat volume, *VFA* visceral fat area

### Quality assessment

The quality assessment is available in Table 2 and shows the study quality ranging from low to high quality, with scores ranging from 4 to 8 on the NOS scale. Eight studies were considered high quality, with a score of 8 [13, 18, 28, 54, 64, 72, 78, 79], fifty-three studies were considered moderate quality, with a score ranging from 5 to 7 [6, 15, 17, 23–27, 29, 30, 32–35, 37–41, 44–49, 51–53, 55–63, 65–71, 73–75, 80–85], and the remaining 9 studies were given a score of 4 and considered low quality [14, 31, 36, 42, 43, 50, 76, 77, 86]. According to the GRADE, the overall quality of the evidence of sarcopenia as a predictive factor for both long-term and short-term should be considered “very low” due to the lack of randomized control trials.

### Long-term outcome assessment

**Fig. 3:**
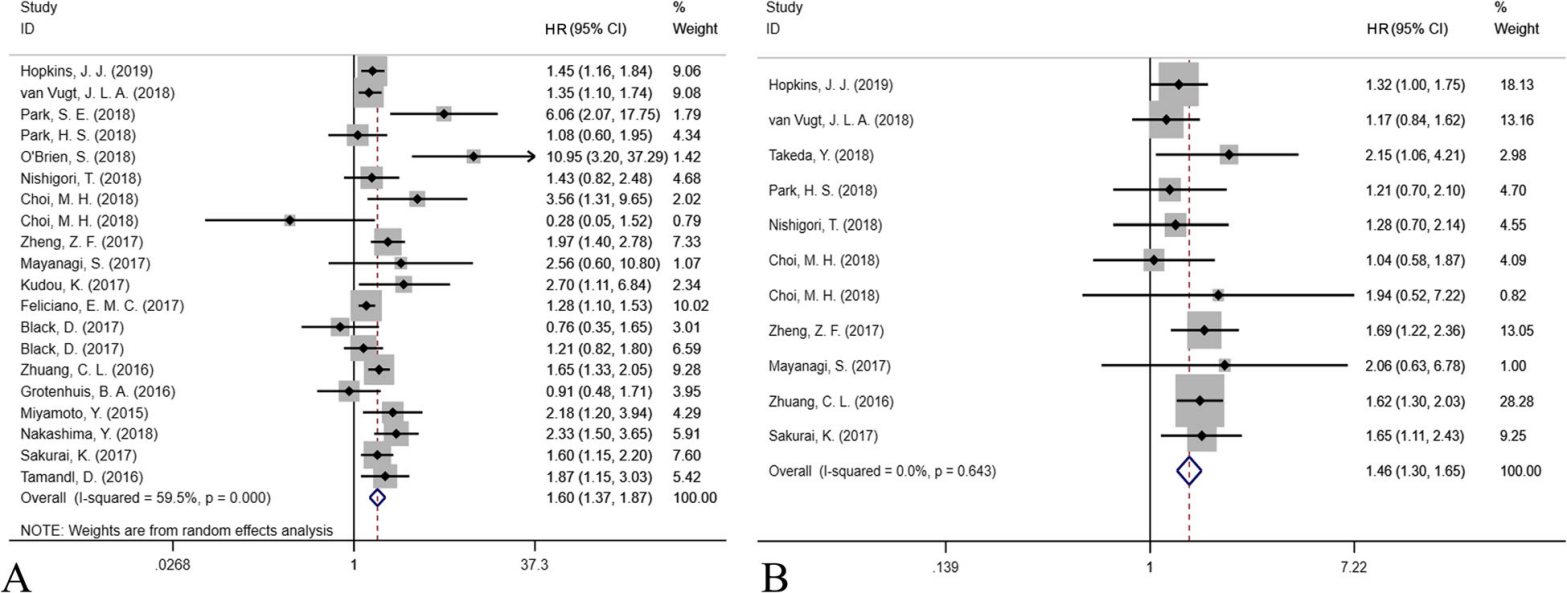
The forest plot for assessing the impact of sarcopenia on long-term outcomes (**a**. overall survival; **b**. disease-free survival)

The forest plot of long-term outcomes after surgery in GI oncology patients is shown in Fig. 3. A total of 20 studies were included for assessing the risk for overall survival (OS) (Fig. 3a), and 11 studies were included for disease-free survival (DFS) (Fig. 3b). The preoperative incidence of sarcopenia was associated both with an increased risk of overall mortality (HR = 1.602, 95% CI = 1.369–1.873, *P* < 0.001, I^2^ = 59.5%, random-effect model) and disease-free mortality (HR = 1.461, 95% CI = 1.297–1.646, P < 0.001, I^2^ = 0%, fixed-effect model).

The subgroup analysis is shown in Table 3. Due to the different body shapes, we compared the Asian countries and non-Asian countries. Moreover, three main criteria and three main diseases, CRC, EC and GC, were compared. In terms of OS, the preoperative incidence of sarcopenia was associated with higher overall mortality in both Asian and non-Asian populations (HR = 1.776 and 1.368, *P* < 0.001 and *P* = 0.002, respectively). Preoperative sarcopenia using cut-offs provided by Zhang et al. and Martin et al. increased the risk of overall mortality (HR = 1.622 and 1.343, respectively, both *P* < 0.001), while no statistically significant increase was observed using the cut-off provided by Prado et al. (HR = 1.976, *P* = 0.075). In terms of the tumor in the three different locations, preoperative sarcopenia was always a risk factor increasing the overall mortality (HR = 1.523, 1.567, and 1.703, *P* = 0.001, 0.015 and < 0.001 in CRC, EC and GC surgical patients, respectively). In terms of DFS, the preoperative incidence of sarcopenia was also associated with a higher risk of disease-free mortality in both Asian and non-Asian populations (*P* < 0.001 and *P* = 0.037, respectively). Similarly, both cut-offs provided by Zhang et al. and Martin et al. were available for defining sarcopenia for predicting the disease-free survival (P < 0.001 and *P* = 0.028). In addition, preoperative sarcopenia was predive for DFS in both CRC and GC surgical patients (*P* = 0.011 and *P* < 0.001, respectively). Only one study focused on EC surgical patients, and this had no statistical significance in predicting DFS (*P* = 0.235).

**Table 3:** The results of subgroup meta-analysis

Subgroup	Cohort	HR or RR	95%CI	P value	I2
Long-term outcome (HR)-Overall survival					
Overall	20	1.602	1.369–1.873	< 0.001	59.5%
Asian countries	12	1.776	1.556–2.026	< 0.001	41.9%
Non-Asian countries	8	1.368	1.117–1.676	0.002	60.1%
Zhuang criteria	2	1.622	1.326–1.983	< 0.001	0%
Martin criteria	5	1.343	1.200–1.503	< 0.001	0%
Prado criteria	6	1.976	0.934–4.182	0.075	74.2%
CRC surgery	7	1.523	1.201–1.930	0.001	63.1%
EC surgery	5	1.567	1.089–2.253	0.015	52.9%
GC surgery	8	1.703	1.281–2.262	< 0.001	60.6%
Long-term outcome (HR)-Disease-free survival					
Overall	11	1.461	1.297–1.646	< 0.001	0%
Asian countries	9	1.566	1.357–1.808	< 0.001	0%
Non-Asian countries	2	1.255	1.014–1.553	0.037	0%
Zhuang criteria	2	1.568	1.274–1.930	< 0.001	0%
Martin criteria	3	1.249	1.024–1.523	0.028	0%
Prado criteria	3	1.271	0.778–2.075	0.338	0%
CRC surgery	4	1.282	1.058–1.554	0.011	0%
EC surgery	1	2.060	0.626–6.783	0.235	–
GC surgery	6	1.578	1.355–1.839	< 0.001	0%
Short-term outcome (RR)-Total complication after surgery					
Overall	15	1.188	1.083–1.303	< 0.001	26.4%
Asian countries	10	1.165	1.046–1.298	0.005	43.9%
Non-Asian countries	5	1.252	1.045–1.499	0.015	0%
Zhuang criteria	4	1.423	1.214–1.667	< 0.001	0%
Martin criteria	5	1.246	1.017–1.527	0.034	0%
Prado criteria	2	1.074	0.842–1.371	0.565	0%
CRC surgery	6	1.314	1.101–1.568	0.002	0%
EC surgery	4	1.051	0.900–1.226	0.531	0%
GC surgery	5	1.218	1.046–1.419	0.011	61.9%
Short-term outcome (RR)-Major complication after surgery					
Overall	14	1.228	1.042–1.448	0.014	12.1%
Asian countries	7	1.244	1.001–1.545	0.049	45.9%
Non-Asian countries	7	1.206	0.936–1.553	0.148	0%
Zhuang criteria	2	2.084	1.359–3.196	0.001	50%
Martin criteria	5	0.988	0.690–1.413	0.946	0%
Prado criteria	4	1.309	0.978–1.750	0.070	0%
CRC surgery	4	0.899	0.545–1.481	0.675	0%
EC surgery	4	1.181	0.932–1.495	0.169	0%
GC surgery	6	1.393	1.076–1.803	0.012	48.3%

*Abbreviation*: *RR* relative risk, *HR* hazard ratio, *CI* confidence interval, *EC* esophageal cancer, *GC* gastric cancer, *CRC* colorectal cancer

### Short-term outcome assessment

**Fig. 4:**
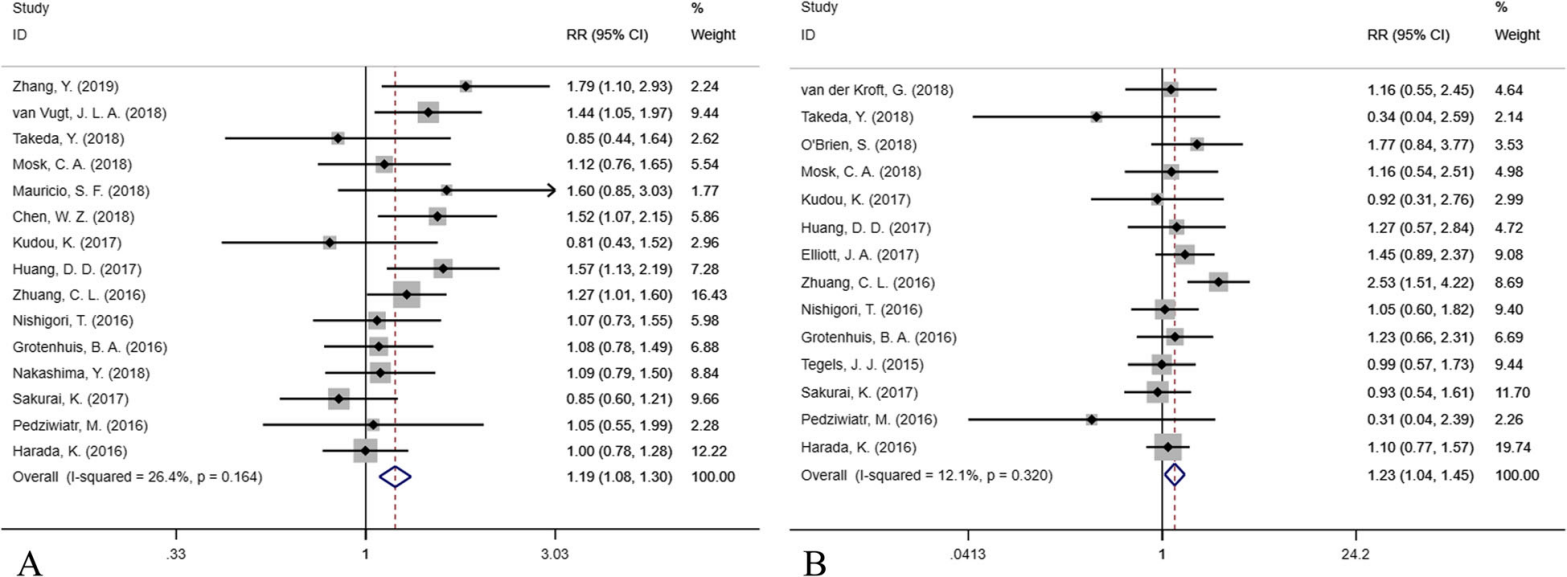
The forest plot in assessing the impact of sarcopenia on short-term outcomes (**a**. total complication; **b**. major complication)

The forest plot of postoperative short-term complications is shown in Fig. 4 (A, total complications; B, major complications). A total of 15 studies reported 1498 postoperative complications occurring in 6489 patients and found that preoperative sarcopenia was a risk factor for total complications (RR = 1.188, 95% CI = 1.083–1.303, P < 0.001, I^2^ = 26.4%, fixed-effect model). Moreover, based on 14 studies with 526 major complications in 4204 patients, the preoperative incidence of sarcopenia was associated with a higher risk of major complications (RR = 1.228, 95% CI = 1.042–1.448, *P* = 0.014, I^2^ = 12.1%, fixed-effect model).

In the subgroup analysis, preoperative sarcopenia was associated with a high risk of total complications in both Asian and non-Asian populations (*P* = 0.005 and *P* = 0.015), while only a slightly significantly higher risk of major complications in Asian populations (*P* = 0.049) and no significantly higher risk in non-Asian populations were found (*P* = 0.148). Preoperative sarcopenia using cut-off provided by Zhuang et al. was a risk factor for increasing both total and major complications after surgery (*P* < 0.001 and *P* = 0.001), while there was no predictive value when using the cut-off from Prado et al. (*P* > 0.05). Sarcopenia was associated with total complications but not any major complication when using the cut-off provided by Martin et al. (*P* = 0.034 and *P* = 0.946). Preoperative sarcopenia was a risk factor in total and major complications after GC surgery (*P* = 0.011 and *P* = 0.012), but not EC surgery (*P* = 0.531 and *P* = 0.169). Sarcopenia was a risk factor for total complications after CRC surgery (*P* = 0.002), but not major complications (*P* = 0.675).

## Discussion

This is the largest-scale systematic review and includes 70 studies to discuss the impact of CT-assessed sarcopenia on GI oncological patients. Our meta-analysis demonstrated that the prevalence of sarcopenia increased with the cut-off of CT-assessed SMI. Preoperative sarcopenia was associated with both long-term outcomes and short-term outcomes. More studies still need to be performed to demonstrate its efficacy in different populations, criteria and diseases.

The impact of nutrition status on oncological patients has been a research hot spot in recent years [33, 84]. Sarcopenia, defined as an age-related muscle reduction disease, has been discussed and updated over time, while the measurement and criteria still need to be determined [23, 87]. It is believed that sarcopenia is a syndrome in which the risk of adverse events is increased with a decrease in skeletal muscle mass associated with decreased muscle strength or function [87]. Because of its objectivity, repeatability, and accuracy, CT is widely used to measure muscle mass, with errors ranging from 1 to 4%, and thus it is considered a “gold standard” [69, 88]. Usually, the L3 muscle area is measured due to its accuracy reflecting the “real” muscle mass and fat volume [89]. Patients undergoing elective GI cancer surgery routinely undergo abdominal CT assessment of the patient’s tumor staging without additional costs. In China, the interval between CT examination and surgery is usually used to optimize the patient’s preoperative status and does not lead to delays in treatment. These optimizations include preoperative nutritional screening and support, physical functioning, pre-rehabilitation, and improvement of comorbidities [90].

Undoubtedly, the incidence of sarcopenia depends mostly on how to define the diagnostic cut-off point for sarcopenia. In our systematic review, a total 19 criteria were used to define sarcopenia. We found that the incidence of SMI was higher when the cut-off of SMI was raised. When using the Western criteria provided by Prado et al. and Martin et al., the incidence of sarcopenia was always higher for the Asian populations, which could be one heterogeneity because of the difference in body shape and diet habit [89, 91]. Although some Asian criteria were proposed, such as by Zhuang et al., Iritani et al. and Kim et al., the validation among countries still needs to be investigated for efficacy and accuracy [13, 92, 93].

Aging is a process in which all functions of the body are declining. Although current research has clarified the relationship between sarcopenia and aging, the specific primary pathogenic factors remain unclear and may be related to a series of changes caused by aging [7, 10]. The number of motor neurons in those over 70 years old is greatly reduced, and skeletal muscle mass begins to shrink at age 30 [12]. Studies have found that sarcopenia is mainly related to the decrease in the number of type II muscle fibers, which is reduced by up to 40% in patients over 70. This could explain why elders are more prone to falls [26]. In our meta-analysis, we demonstrated that preoperative sarcopenia might increase by 1.1–1.2-fold the risk of total and major complications in GI patients. Patients suffering from sarcopenia may feel weak, with limited mobility, which in turn affects the postoperative recovery process. However, until now, there was no evidence to demonstrate that preoperative increase in muscle mass could improve the outcome of GI oncological patients. One reason is that the short period during cancer diagnosis and surgery might not be enough to improve nutritional status. Most older people have insufficient protein intake or absorption barriers. Moreover, with the nutrition consumption in tumors, malnutrition and weight loss are common problems in GI malignancy patients. It not only affects hospitalization time and costs but also affects the quality of life and long-term survival of patients. Therefore, preoperative sarcopenia may be associated with postoperative complications. Early identification of the onset of sarcopenia in the elderly population and early intervention may help the patient maintain muscle mass and improve patient outcomes during treatment. Nutritional support therapy can improve the prognosis of hospitalized patients, but there is controversy about the improvement of muscle mass and function, while exercise is beneficial in the maintenance of human physiological functions [94]. The American Cancer Society (ACS) has recommended clinical activities for all cancer patients based on clinical research on aerobic exercise and resistance training in recent decades. Age-related muscle mass and muscle strength reduction also depend on individual health status, heredity, activity function, muscle mass and muscle strength training, and nutritional levels [12]. Patients with sedentary movements have a more pronounced decrease in the number and intensity of muscle fibers compared with patients with normal activities, revealing that exercise can slow muscle atrophy. Active exercise combined with essential amino acid nutrition support can improve muscle status and is an effective way to fight muscle deficiency [95].

Most studies currently focus only on the relationship between sarcopenia and clinical outcomes and rarely explore the causes. Studies have found that muscle reduction reflects an increase in the metabolism of malignant tumors, resulting in an increased systemic inflammatory response and increased muscle consumption [96]. Moreover, several studies found that a systemic inflammatory response significantly increased the adverse outcomes of patients [97, 98]. Richards et al. found a clear correlation between muscle reduction in patients with resectable primary CRC and systemic inflammatory response [97]. Aleman et al. suggested that inflammatory cells may participate in the onset of sarcopenia by interfering with the skeletal muscle insulin-like growth factor-I pathway [98]. This may explain why the poor prognosis in sarcopenia may be related to an increase in the systemic inflammatory response. Sarcopenia may also be affected by genetic factors. A genome-wide association study found that genes associated with sarcopenia and osteoporosis include growth differentiation factor 8, myocyte enhancer factor 2C, and peroxisome proliferator receptor gamma coactivator 1a. There are currently few reports on the genetics of sarcopenia, and further research is still needed [99].

There were some limitations to our study. First, due to the observational nature of the available studies, the evidence was “low quality” by the GRADE criteria. More prospective randomized control trials need to be performed to investigate the efficacy of sarcopenia in predicting outcomes in oncological patients. Second, due to the heterogeneity existing due to the different cut-offs and diseases, the included studies had few subgroup analyses. Third, the Clavien-Dindo classification is suitable for assessing postoperative complications, while sarcopenia may be associated with some specific complications, such as respiratory complications, infectious complications and postoperative leakage, which could not be calculated in every included study. Further efforts need to be made in individual patient meta-analyses and regressions to discuss the risk of sarcopenia in oncological patients.

## Conclusion

The prevalence of sarcopenia increases when the cut-off of SMI increases. The preoperative incidence of sarcopenia is a risk factor for both overall and disease-free survival and short-term total and major postoperative complications in the whole population of gastrointestinal oncology patients. In the subgroup analysis, sarcopenia is related to higher complication, recurrence and mortality rates in CRC and GC surgical patients. The cut-off provided by Martin et al. is the most common predictable criteria globally, while more Western cohorts need to be validated when using cut-offs provided by Asian countries.

## Data Availability

Not applicable.

## Abbreviations

CI: Confidence interval
CRC: Colorectal cancer
CT: Computed tomography
DFS: Disease-free survival
EC: Esophageal cancer
GC: Gastric cancer
GI: Gastrointestinal
GRADE: The grading of recommendations, assessment, development, and evaluation
HR: Hazard ratio
L3: The third lumbar
OS: Overall survival
PRISMA: Preferred reporting items for systematic review and meta-analysis
RR: Relative risk
SMI: Skeletal muscle index
TPA: Total psoas area
VFA: Visceral fat area
VFV: Visceral fat volume

## References

[1] Ferlay J, Soerjomataram I, Dikshit R, Eser S, Mathers C, Rebelo M, Parkin DM, Forman D, Bray F (2015). Cancer incidence and mortality worldwide: sources, methods and major patterns in GLOBOCAN 2012. Int J Cancer.

[2] Siegel RL, Miller KD, Jemal A (2016). Cancer statistics, 2016. CA Cancer J Clin.

[3] Mislang AR, Di Donato S, Hubbard J, Krishna L, Mottino G, Bozzetti F, Biganzoli L (2018). Nutritional management of older adults with gastrointestinal cancers: an International Society of Geriatric Oncology (SIOG) review paper. J Geriatr Oncol.

[4] Smyth E, Verheij M, Allum W, Cunningham D, Cervantes A, Arnold D (2016). Gastric cancer: ESMO Clinical Practice Guidelines for diagnosis, treatment and follow-up. Ann Oncol.

[5] Fukuda Y, Yamamoto K, Hirao M, Nishikawa K, Nagatsuma Y, Nakayama T, Tanikawa S, Maeda S, Uemura M, Miyake M (2016). Sarcopenia is associated with severe postoperative complications in elderly gastric cancer patients undergoing gastrectomy. Gastric Cancer.

[6] van der Kroft G, Bours DMJL, Janssen-Heijnen DM, van Berlo DCLH, Konsten DJLM (2018). Value of sarcopenia assessed by computed tomography for the prediction of postoperative morbidity following oncological colorectal resection: a comparison with the malnutrition screening tool. Clinical Nutrition ESPEN.

[7] Rosenberg I (1989). Epidemiologic and methodologic problems in determining nutritional status of older persons. (summary comments). Am J Cli Nutr.

[8] Sousa AS, Guerra RS, Fonseca I, Pichel F, Amaral TF (2015). Sarcopenia among hospitalized patients–a cross-sectional study. Clin Nutr.

[9] Ishii S, Tanaka T, Shibasaki K, Ouchi Y, Kikutani T, Higashiguchi T, Obuchi SP, Ishikawa-Takata K, Hirano H, Kawai H (2014). Development of a simple screening test for sarcopenia in older adults. Geriatr Gerontol Int.

[10] Paddon-Jones D, Short KR, Campbell WW, Volpi E, Wolfe RR (2008). Role of dietary protein in the sarcopenia of aging. Am J Clin Nutr.

[11] Sayer AA, Dennison EM, Syddall HE, Jameson K, Martin HJ, Cooper C (2008). The developmental origins of sarcopenia: using peripheral quantitative computed tomography to assess muscle size in older people. J Gerontol Ser A Biol Med Sci.

[12] von Haehling S, Morley JE, Anker SD (2010). An overview of sarcopenia: facts and numbers on prevalence and clinical impact. J Cachexia Sarcopenia Muscle.

[13] Zhuang CL, Huang DD, Pang WY, Zhou CJ, Wang SL, Lou N, Ma LL, Yu Z, Shen X (2016). Sarcopenia is an independent predictor of severe postoperative complications and long-term survival after radical Gastrectomy for gastric Cancer: analysis from a large-scale cohort. Medicine.

[14] Heus C, Cakir H, Lak A, Doodeman HJ, Houdijk AP (2016). Visceral obesity, muscle mass and outcome in rectal cancer surgery after neo-adjuvant chemo-radiation. Int J Surg.

[15] Park BK, Park JW, Ryoo SB, Jeong SY, Park KJ, Park JG (2015). Effect of visceral obesity on surgical outcomes of patients undergoing laparoscopic colorectal surgery. World J Surg.

[16] Shachar SS, Williams GR, Muss HB, Nishijima TF (2016). Prognostic value of sarcopenia in adults with solid tumours: a meta-analysis and systematic review. Eur J Cancer.

[17] Ouchi A, Asano M, Aono K, Watanabe T, Oya S (2016). Laparoscopic colorectal resection in patients with sarcopenia: a retrospective case-control study. J Laparoendoscopic Adv Surg Techniques Part A.

[18] Sakurai K, Kubo N, Tamura T, Toyokawa T, Amano R, Tanaka H, Muguruma K, Yashiro M, Maeda K, Hirakawa K (2017). Adverse effects of low preoperative skeletal muscle mass in patients undergoing Gastrectomy for gastric Cancer. Ann Surg Oncol.

[19] Moher D, Liberati A, Tetzlaff J, Altman DG, Group P (2009). Preferred reporting items for systematic reviews and meta-analyses: the PRISMA statement. PLoS Med.

[20] Clavien PA, Barkun J, De Oliveira ML, Vauthey JN, Dindo D, Schulick RD, De Santibañes E, Pekolj J, Slankamenac K, Bassi C (2009). The Clavien-Dindo classification of surgical complications: five-year experience. Ann Surg.

[21] Stang A (2010). Critical evaluation of the Newcastle-Ottawa scale for the assessment of the quality of nonrandomized studies in meta-analyses. Eur J Epidemiol.

[22] Balshem H, Helfand M, Schünemann HJ, Oxman AD, Kunz R, Brozek J, Vist GE, Falck-Ytter Y, Meerpohl J, Norris S (2011). GRADE guidelines: 3. Rating the quality of evidence. J Clin Epidemiol.

[23] Yang J., Zhang T., Feng D., Dai X., Lv T., Wang X., Gong J., Zhu W., Li J. (2019). A new diagnostic index for sarcopenia and its association with short‐term postoperative complications in patients undergoing surgery for colorectal cancer. Colorectal Disease.

[24] Hopkins JJ, Reif RL, Bigam DL, Baracos VE, Eurich DT, Sawyer MB (2019). The impact of muscle and adipose tissue on long-term survival in patients with stage I to III colorectal Cancer. Dis Colon Rectum.

[25] Zhang Y, Wang JP, Wang XL, Tian H, Gao TT, Tang LM, Tian F, Wang JW, Zheng HJ, Zhang L, et al. Computed tomography-quantified body composition predicts short-term outcomes after gastrectomy in gastric cancer. Curr Oncol. 2018;25(5):e411–22. 10.3747/co.25.4014 PMC6209549 30464692

[26] Zhang WT, Lin J, Chen WS, Huang YS, Wu RS, Chen XD, Lou N, Chi CH, Hu CY, Shen X (2018). Sarcopenic obesity is associated with severe postoperative complications in gastric Cancer patients undergoing Gastrectomy: a prospective study. J Gastrointest Surg.

[27] Wang SL, Ma LL, Chen XY, Zhou DL, Li B, Huang DD, Yu Z, Shen X, Zhuang CL (2018). Impact of visceral fat on surgical complications and long-term survival of patients with gastric cancer after radical gastrectomy. Eur J Clin Nutr.

[28] van Vugt JLA, van den Braak RRJ C, Lalmahomed ZS, Vrijland WW, JWT D, DDE Z, Vles WJ, PPLO C, IJ JNM (2018). Impact of low skeletal muscle mass and density on short and long-term outcome after resection of stage I-III colorectal cancer. Eur J Surg Oncol.

[29] Takeda Y, Akiyoshi T, Matsueda K, Fukuoka H, Ogura A, Miki H, Hiyoshi Y, Nagasaki T, Konishi T, Fujimoto Y (2018). Skeletal muscle loss is an independent negative prognostic factor in patients with advanced lower rectal cancer treated with neoadjuvant chemoradiotherapy. PLoS One.

[30] Sugiyama K, Narita Y, Mitani S, Honda K, Masuishi T, Taniguchi H, Kadowaki S, Ura T, Ando M, Tajika M (2018). Baseline sarcopenia and skeletal muscle loss during chemotherapy affect survival outcomes in metastatic gastric Cancer. Anticancer Res.

[31] Souza BU, Souza NCS, Martucci RB, Rodrigues VD, Pinho NB, Gonzalez MC, Avesani CM (2018). Factors associated with sarcopenia in patients with colorectal Cancer. Nutr Cancer.

[32] Park SE, Hwang IG, Choi CH, Kang H, Kim BG, Park BK, Cha SJ, Jang JS, Choi JH (2018). Sarcopenia is poor prognostic factor in older patients with locally advanced rectal cancer who received preoperative or postoperative chemoradiotherapy. Medicine.

[33] Park HS, Kim HS, Beom SH, Rha SY, Chung HC, Kim JH, Chun YJ, Lee SW, Choe EA, Heo SJ (2018). Marked loss of muscle, visceral fat, or subcutaneous fat after Gastrectomy predicts poor survival in advanced gastric Cancer: single-center study from the CLASSIC trial. Ann Surg Oncol.

[34] O'Brien S, Twomey M, Moloney F, Kavanagh RG, Carey BW, Power D, Maher MM, O'Connor OJ, O'Suilleabhain C (2018). Sarcopenia and post-operative morbidity and mortality in patients with gastric Cancer. J Gastric Cancer.

[35] Nishigori T, Tsunoda S, Obama K, Hisamori S, Hashimoto K, Itatani Y, Okada K, Sakai Y (2018). Optimal cutoff values of skeletal muscle index to define sarcopenia for prediction of survival in patients with advanced gastric Cancer. Ann Surg Oncol.

[36] Nipp RD, Fuchs G, El-Jawahri A, Mario J, Troschel FM, Greer JA, Gallagher ER, Jackson VA, Kambadakone A, Hong TS (2018). Sarcopenia is associated with quality of life and depression in patients with advanced Cancer. Oncologist.

[37] Mosk CA, van Vugt JLA, de Jonge H, Witjes CDM, Buettner S, Ijzermans JNM, van der Laan L (2018). Low skeletal muscle mass as a risk factor for postoperative delirium in elderly patients undergoing colorectal cancer surgery. Clin Interv Aging.

[38] Mauricio SF, Xiao J, Prado CM, Gonzalez MC, Correia MITD (2018). Different nutritional assessment tools as predictors of postoperative complications in patients undergoing colorectal cancer resection. Clin Nutr.

[39] Martin L, Hopkins J, Malietzis G, Jenkins JT, Sawyer MB, Brisebois R, MacLean A, Nelson G, Gramlich L, Baracos VE (2018). Assessment of computed tomography (CT)-defined muscle and adipose tissue features in relation to Short-term outcomes after elective surgery for colorectal Cancer: a multicenter approach. Ann Surg Oncol.

[40] Mao CC, Chen XD, Lin J, Zhu-Ge WS, Xie ZD, Chen XY, Zhang FM, Wu RS, Zhang WT, Lou N (2018). A novel Nomogram for predicting postsurgical intra-abdominal infection in gastric Cancer patients: a prospective study. J Gastrointest Surg.

[41] Lin J, Zhang W, Huang Y, Chen W, Wu R, Chen X, Lou N, Wang P (2018). Sarcopenia is associated with the neutrophil/ lymphocyte and platelet/lymphocyte ratios in operable gastric cancer patients: a prospective study. Cancer Manag Res.

[42] Kurk SA, Peeters PHM, Dorresteijn B, de Jong PA, Jourdan M, Kuijf HJ, Punt CJA, Koopman M, May AM (2018). Impact of different palliative systemic treatments on skeletal muscle mass in metastatic colorectal cancer patients. J Cachexia Sarcopenia Muscle.

[43] Guinan EM, Doyle SL, Bennett AE, O'Neill L, Gannon J, Elliott JA, O'Sullivan J, Reynolds JV, Hussey J (2018). Sarcopenia during neoadjuvant therapy for oesophageal cancer: characterising the impact on muscle strength and physical performance. Support Care Cancer.

[44] Choi MH, Oh SN, Lee IK, Oh ST, Won DD (2018). Sarcopenia is negatively associated with long-term outcomes in locally advanced rectal cancer. J Cachexia Sarcopenia Muscle.

[45] Choi MH, Kim KA, Hwang SS, Byun JY (2018). CT-quantified muscle and fat change in patients after surgery or endoscopic resection for early gastric cancer and its impact on long-term outcomes. Medicine.

[46] Chen WZ, Chen XD, Ma LL, Zhang FM, Lin J, Zhuang CL, Yu Z, Chen XL, Chen XX (2018). Impact of visceral obesity and sarcopenia on Short-term outcomes after colorectal Cancer surgery. Dig Dis Sci.

[47] Beuran M, Tache C, Ciubotaru C, Vartic M, Hostiuc S, Prodan A, Sartelli M, Griffiths EA, Hernandez M, Negoi I (2018). Sarcopenia is a Predictive Factor for Postoperative Morbidity and Mortality in Patients Having Radical Gastrectomy for Cancer. Chirurgia.

[48] Zhou CJ, Zhang FM, Zhang FY, Yu Z, Chen XL, Shen X, Zhuang CL, Chen XX (2017). Sarcopenia: a new predictor of postoperative complications for elderly gastric cancer patients who underwent radical gastrectomy. J Surg Res.

[49] Zheng ZF, Lu J, Zheng CH, Li P, Xie JW, Wang JB, Lin JX, Chen QY, Lin M, Huang CM (2017). A novel prognostic scoring system based on preoperative sarcopenia predicts the long-term outcome for patients after R0 resection for gastric Cancer: experiences of a high-volume center. Ann Surg Oncol.

[50] Palmela C, Velho S, Agostinho L, Branco F, Santos M, Santos MP, Oliveira MH, Strecht J, Maio R, Cravo M (2017). Body composition as a prognostic factor of Neoadjuvant chemotherapy toxicity and outcome in patients with locally advanced gastric Cancer. J Gastric Cancer.

[51] Mirkin KA, Luke FE, Gangi A, Pimiento JM, Jeong D, Hollenbeak CS, Wong J (2017). Sarcopenia related to neoadjuvant chemotherapy and perioperative outcomes in resected gastric cancer: a multiinstitutional analysis. J Gastrointes Oncol.

[52] Mayanagi S, Tsubosa Y, Omae K, Niihara M, Uchida T, Tsushima T, Yokota T, Sato H, Naito T, Yasui H (2017). Negative impact of skeletal muscle wasting after Neoadjuvant chemotherapy followed by surgery on survival for patients with thoracic esophageal Cancer. Ann Surg Oncol.

[53] Lou N, Chi CH, Chen XD, Zhou CJ, Wang SL, Zhuang CL, Shen X (2017). Sarcopenia in overweight and obese patients is a predictive factor for postoperative complication in gastric cancer: a prospective study. Eur J Surg Oncol.

[54] Kudou K, Saeki H, Nakashima Y, Edahiro K, Korehisa S, Taniguchi D, Tsutsumi R, Nishimura S, Nakaji Y, Akiyama S (2017). Prognostic significance of sarcopenia in patients with Esophagogastric junction Cancer or upper gastric Cancer. Ann Surg Oncol.

[55] Huang DD, Zhou CJ, Wang SL, Mao ST, Zhou XY, Lou N, Zhang Z, Yu Z, Shen X, Zhuang CL (2017). Impact of different sarcopenia stages on the postoperative outcomes after radical gastrectomy for gastric cancer. Surgery.

[56] Feliciano EMC, Kroenke CH, Meyerhardt JA, Prado CM, Bradshaw PT, Kwan ML, Xiao J, Alexeeff S, Corley D, Weltzien E (2017). Association of Systemic Inflammation and Sarcopenia with Survival in nonmetastatic colorectal Cancer: results from the C SCANS study. JAMA Oncology.

[57] Elliott JA, Doyle SL, Murphy CF, King S, Guinan EM, Beddy P, Ravi N, Reynolds JV (2017). Sarcopenia: prevalence, and impact on operative and oncologic outcomes in the multimodal Management of Locally Advanced Esophageal Cancer. Ann Surg.

[58] Black D, Mackay C, Ramsay G, Hamoodi Z, Nanthakumaran S, Park KGM, Loudon MA, Richards CH (2017). Prognostic value of computed tomography: measured parameters of body composition in primary operable gastrointestinal cancers. Ann Surg Oncol.

[59] Wang SL, Zhuang CL, Huang DD, Pang WY, Lou N, Chen FF, Zhou CJ, Shen X, Yu Z (2016). Sarcopenia adversely impacts postoperative clinical outcomes following Gastrectomy in patients with gastric Cancer: a prospective study. Ann Surg Oncol.

[60] Takeuchi M, Ishii K, Seki H, Yasui N, Sakata M, Shimada A, Matsumoto H (2016). Excessive visceral fat area as a risk factor for early postoperative complications of total gastrectomy for gastric cancer: a retrospective cohort study. BMC Surg.

[61] Nishigori T, Okabe H, Tanaka E, Tsunoda S, Hisamori S, Sakai Y (2016). Sarcopenia as a predictor of pulmonary complications after esophagectomy for thoracic esophageal cancer. J Surg Oncol.

[62] Malietzis G, Currie AC, Athanasiou T, Johns N, Anyamene N, Glynne-Jones R, Kennedy RH, Fearon KC, Jenkins JT (2016). Influence of body composition profile on outcomes following colorectal cancer surgery. Br J Surg.

[63] Huang DD, Chen XX, Chen XY, Wang SL, Shen X, Chen XL, Yu Z, Zhuang CL (2016). Sarcopenia predicts 1-year mortality in elderly patients undergoing curative gastrectomy for gastric cancer: a prospective study. J Cancer Res Clin Oncol.

[64] Grotenhuis BA, Shapiro J, van Adrichem S, de Vries M, Koek M, Wijnhoven BP, van Lanschot JJ (2016). Sarcopenia/muscle mass is not a prognostic factor for Short- and long-term outcome after Esophagectomy for Cancer. World J Surg.

[65] Chemama S, Bayar MA, Lanoy E, Ammari S, Stoclin A, Goere D, Elias D, Raynard B, Antoun S (2016). Sarcopenia is associated with chemotherapy toxicity in patients undergoing Cytoreductive surgery with Hyperthermic Intraperitoneal chemotherapy for peritoneal Carcinomatosis from colorectal Cancer. Ann Surg Oncol.

[66] Blauwhoff-Buskermolen S, Versteeg KS, de van der Schueren MA, den Braver NR, Berkhof J, Langius JA, Verheul HM (2016). Loss of muscle mass during chemotherapy is predictive for poor survival of patients with metastatic colorectal Cancer. J Clin Oncol.

[67] Anandavadivelan P, Brismar TB, Nilsson M, Johar AM, Martin L (2016). Sarcopenic obesity: a probable risk factor for dose limiting toxicity during neo-adjuvant chemotherapy in oesophageal cancer patients. Clin Nutr.

[68] Tegels JJ, van Vugt JL, Reisinger KW, Hulsewe KW, Hoofwijk AG, Derikx JP, Stoot JH (2015). Sarcopenia is highly prevalent in patients undergoing surgery for gastric cancer but not associated with worse outcomes. J Surg Oncol.

[69] Reisinger KW, van Vugt JL, Tegels JJ, Snijders C, Hulsewe KW, Hoofwijk AG, Stoot JH, Von Meyenfeldt MF, Beets GL, Derikx JP (2015). Functional compromise reflected by sarcopenia, frailty, and nutritional depletion predicts adverse postoperative outcome after colorectal cancer surgery. Ann Surg.

[70] Miyamoto Y, Baba Y, Sakamoto Y, Ohuchi M, Tokunaga R, Kurashige J, Hiyoshi Y, Iwagami S, Yoshida N, Yoshida M (2015). Sarcopenia is a negative prognostic factor after curative resection of colorectal Cancer. Ann Surg Oncol.

[71] Li XT, Tang L, Chen Y, Li YL, Zhang XP, Sun YS (2015). Visceral and subcutaneous fat as new independent predictive factors of survival in locally advanced gastric carcinoma patients treated with neo-adjuvant chemotherapy. J Cancer Res Clin Oncol.

[72] Huang DD, Wang SL, Zhuang CL, Zheng BS, Lu JX, Chen FF, Zhou CJ, Shen X, Yu Z (2015). Sarcopenia, as defined by low muscle mass, strength and physical performance, predicts complications after surgery for colorectal cancer. Color Dis.

[73] Yip C, Goh V, Davies A, Gossage J, Mitchell-Hay R, Hynes O, Maisey N, Ross P, Gaya A, Landau DB (2014). Assessment of sarcopenia and changes in body composition after neoadjuvant chemotherapy and associations with clinical outcomes in oesophageal cancer. Eur Radiol.

[74] Barret M, Antoun S, Dalban C, Malka D, Mansourbakht T, Zaanan A, Latko E, Taieb J (2014). Sarcopenia is linked to treatment toxicity in patients with metastatic colorectal cancer. Nutr Cancer.

[75] Lieffers JR, Fassbender K, Winget M, Baracos VE, Bathe OF (2012). Sarcopenia is associated with postoperative infection and delayed recovery from colorectal cancer resection surgery. Br J Cancer.

[76] Awad S, Tan BH, Cui H, Bhalla A, Fearon KC, Parsons SL, Catton JA, Lobo DN (2012). Marked changes in body composition following neoadjuvant chemotherapy for oesophagogastric cancer. Clin Nutr.

[77] Guiu B, Petit JM, Bonnetain F, Ladoire S, Guiu S, Cercueil JP, Krause D, Hillon P, Borg C, Chauffert B (2010). Visceral fat area is an independent predictive biomarker of outcome after first-line bevacizumab-based treatment in metastatic colorectal cancer. Gut.

[78] Nakashima Y, Saeki H, Nakanishi R, Sugiyama M, Kurashige J, Oki E, Maehara Y (2018). Assessment of sarcopenia as a predictor of poor outcomes after Esophagectomy in elderly patients with esophageal Cancer. Ann Surg.

[79] Paireder M, Asari R, Kristo I, Rieder E, Tamandl D, Ba-Ssalamah A, Schoppmann SF (2017). Impact of sarcopenia on outcome in patients with esophageal resection following neoadjuvant chemotherapy for esophageal cancer. Eur J Surg Oncol.

[80] Tamandl D, Paireder M, Asari R, Baltzer PA, Schoppmann SF, Ba-Ssalamah A (2016). Markers of sarcopenia quantified by computed tomography predict adverse long-term outcome in patients with resected oesophageal or gastro-oesophageal junction cancer. Eur Radiol.

[81] Pedziwiatr M, Pisarska M, Major P, Grochowska A, Matlok M, Przeczek K, Stefura T, Budzynski A, Klek S (2016). Laparoscopic colorectal cancer surgery combined with enhanced recovery after surgery protocol (ERAS) reduces the negative impact of sarcopenia on short-term outcomes. Eur J Surg Oncol.

[82] Hayashi N, Ando Y, Gyawali B, Shimokata T, Maeda O, Fukaya M, Goto H, Nagino M, Kodera Y (2016). Low skeletal muscle density is associated with poor survival in patients who receive chemotherapy for metastatic gastric cancer. Oncol Rep.

[83] Harada K, Ida S, Baba Y, Ishimoto T, Kosumi K, Tokunaga R, Izumi D, Ohuchi M, Nakamura K, Kiyozumi Y (2016). Prognostic and clinical impact of sarcopenia in esophageal squamous cell carcinoma. Dis Esophagus.

[84] Chen FF, Zhang FY, Zhou XY, Shen X, Yu Z, Zhuang CL (2016). Role of frailty and nutritional status in predicting complications following total gastrectomy with D2 lymphadenectomy in patients with gastric cancer: a prospective study. Langenbeck's Arch Surg.

[85] Tan BH, Brammer K, Randhawa N, Welch NT, Parsons SL, James EJ, Catton JA (2015). Sarcopenia is associated with toxicity in patients undergoing neo-adjuvant chemotherapy for oesophago-gastric cancer. Eur J Surg Oncol.

[86] Jones KI, Doleman B, Scott S, Lund JN, Williams JP (2015). Simple psoas cross-sectional area measurement is a quick and easy method to assess sarcopenia and predicts major surgical complications. Colorectal Dis.

[87] Chen L-K, Liu L-K, Woo J, Assantachai P, Auyeung T-W, Bahyah KS, Chou M-Y, Chen L-Y, Hsu P-S, Krairit O (2014). Sarcopenia in Asia: consensus report of the Asian working Group for Sarcopenia. J Am Med Dir Assoc.

[88] Mitsiopoulos N, Baumgartner R, Heymsfield S, Lyons W, Gallagher D, Ross R (1998). Cadaver validation of skeletal muscle measurement by magnetic resonance imaging and computerized tomography. J Appl Physiol.

[89] Prado CM, Lieffers JR, McCargar LJ, Reiman T, Sawyer MB, Martin L, Baracos VE (2008). Prevalence and clinical implications of sarcopenic obesity in patients with solid tumours of the respiratory and gastrointestinal tracts: a population-based study. Lancet Oncol.

[90] Fearon K, Arends J, Baracos V (2013). Understanding the mechanisms and treatment options in cancer cachexia. Nat Rev Clin Oncol.

[91] Martin L, Birdsell L, Macdonald N, Reiman T, Clandinin MT, McCargar LJ, Murphy R, Ghosh S, Sawyer MB, Baracos VE (2013). Cancer cachexia in the age of obesity: skeletal muscle depletion is a powerful prognostic factor, independent of body mass index. J Clin Oncol.

[92] Kim EY, Kim YS, Park I, Ahn HK, Cho EK, Jeong YM (2015). Prognostic significance of CT-determined sarcopenia in patients with small-cell lung cancer. J Thorac Oncol.

[93] Iritani S, Imai K, Takai K, Hanai T, Ideta T, Miyazaki T, Suetsugu A, Shiraki M, Shimizu M, Moriwaki H (2015). Skeletal muscle depletion is an independent prognostic factor for hepatocellular carcinoma. J Gastroenterol.

[94] Visser M, Pluijm SM, Stel VS, Bosscher RJ, Deeg DJ (2002). Physical activity as a determinant of change in mobility performance: the longitudinal aging study Amsterdam. J Am Geriatr Soc.

[95] Law WL, Choi HK, Lee YM, Ho JW (2007). The impact of postoperative complications on long-term outcomes following curative resection for colorectal cancer. Ann Surg Oncol.

[96] Dodson S, Baracos VE, Jatoi A, Evans WJ, Cella D, Dalton JT, Steiner MS (2011). Muscle wasting in cancer cachexia: clinical implications, diagnosis, and emerging treatment strategies. Annu Rev Med.

[97] Richards CH, Roxburgh CS, MacMillan MT, Isswiasi S, Robertson EG, Guthrie GK, Horgan PG, McMillan DC (2012). The relationships between body composition and the systemic inflammatory response in patients with primary operable colorectal cancer. PLoS One.

[98] Alemán MR, Santolaria F, Batista N, Marıa J, González-Reimers E, Milena A, Llanos M, Gómez-Sirvent JL (2002). Leptin role in advanced lung cancer. A mediator of the acute phase response or a marker of the status of nutrition?. Cytokine.

[99] Karasik D, Zinder M (2012). The genetic pleiotropy of musculoskeletal aging. Front Physiol.

